# Discovery of tauroursodeoxycholic acid biotransformation enzymes from the gut microbiome of black bears using metagenomics

**DOI:** 10.1038/srep45495

**Published:** 2017-04-24

**Authors:** Can Song, Bochu Wang, Jun Tan, Liancai Zhu, Deshuai Lou

**Affiliations:** 1 Key Laboratory of Biorheological Science and Technology (Chongqing University), Ministry of Education, College of Bioengineering, Chongqing University, Chongqing 400030, China; 2 Chongqing Key Laboratory of Medicinal Resources in the Three Gorges Reservoir Region, School of Biological & Chemical engineering, Chongqing University of Education, Chongqing 400067, China

## Abstract

Tauroursodeoxycholic acid (TUDCA) has been used to treat many diseases effectively. 7α-hydroxysteroid dehydrogenase (7α-HSDH) and 7β-hydroxysteroid dehydrogenase (7β-HSDH) are two key enzymes that drive the efficient biosynthesis of TUDCA from taurochenodeoxycholic acid (TCDCA) *in vitro*. In this study, a metagenomic approach was used to isolate 7α- and 7β-HSDHs from fecal samples of black bears. Five new 7α-HSDHs and one new 7β-HSDH enzyme were discovered and identified from the gut microbiota of black bears, and four of them presented good enzymatic properties. Our data also suggest cooperation in the biotransformation of TUDCA by the gut microbiota in black bears. In conclusion, this work expands the natural enzyme bank of HSDHs, provides promising candidate enzymes for application in the biosynthesis TUDCA and the epimerization reaction of bile acids at the C-7 position, and provides a data set for the discovery of novel enzymes in the gut micriobiome of black bears.

Animal bile has been used in traditional Chinese medicine for thousands of years because of its pharmaceutical potential function and value for unique clinical applications^1^. Both free and conjugated bile acids are natural products and fundamental components of bile, which plays an important role in the pathways of human fat metabolism.

Tauroursodeoxycholic acid (TUDCA), a kind of conjugated bile acid and the physiologically active form of ursodeoxycholic acid (UDCA), has various pharmaceutical applications due to its beneficial effects on the treatment of hepatobiliary disease^2^,^3^,^4^, improving therapy effects in Alzheimer’s disease and Parkinson’s disease^5^,^6^, and preventing apoptosis-related diseases^7^,^8^. Taurochenodeoxycholic acid (TCDCA) is a precursor of TUDCA, and the two constitute an isomers pair^9^. *In vivo*, TUDCA is synthesized by intestinal microorganisms in several steps via hydroxysteroid dehydrogenases (HSDHs), members of the short-chain dehydrogenase/reductase (SDR) superfamily^10^,^11^,^12^. *In vitro*, TUDCA can be directly biosynthesized from TCDCA in a two-step enzymatic reaction (Fig. 1A): First, TCDCA is oxidized to taurine-7-ketolithocholic acid (T7-KLCA) via 7α-HSDH, coupled to the regeneration of the cofactor NAD^+^. Then, T7-KLCA is reduced to TUDCA by 7β-HSDH, accompanied by the regeneration of the cofactor NADPH. In the past, TUDCA was prepared by chemical synthesis^13^, and compared to chemical epimerization, biotransformation of TUDCA from TCDCA is a mild and environmentally friendly process.

Therefore, 7α-HSDHs and 7β-HSDHs are two key enzymes for the efficient biotransformation of TCDCA to TUDCA. Genes encoding 7α-HSDHs have been cloned from *Eubacterium sp.*^14^, *Escherichia coli*^15^, *Clostridium sordellii*^16^, *Bacteroides fragilis*^17^ and *Clostridium absonum*^18^. In contrast, less information is available for 7β-HSDHs. So far, four genes encoding 7β-HSDHs have been cloned from *C. absonum*^18^, *Collinsella aerofaciens*^19^, *Ruminococcus gnavus*^20^ and *R. torques*^21^. Thus, it is necessary to find more and better 7α-HSDHs and 7β-HSDHs for the efficient biosynthesis of TUDCA.

The Asiatic black bear (*Ursus thibetanus*), also called the moon bear or white-chested bear, is the only mammal known to produce significant amounts of TUDCA. Therefore, the gut microbiome of black bears provide a great resource to seek more and better 7α-HSDHs and 7β-HSDHs. In this study, we used a metagenomic approach, discovered six HSDH encoding genes (five 7α-HSDH genes and one 7β-HSDH gene) in the gut microbiome of black bears, and subsequently characterized their functions and enzymatic properties.

## Materials and Methods

### Ethic Statement and sample collection

Before sample collection, all animal work was approved by the Institutional Animal Care and Use Committee of the Chongqing University under permit number CBE-A20140702. All experiments were performed in accordance with the approved guidelines and regulations.

Fecal samples from captive black bears were collected immediately after their natural defecation, immediately frozen by liquid nitrogen and sent to the laboratory in foam boxes with dry ice. All samples were obtained from the inside of the feces using sterilized equipment, in which there was no contact with soil or other pollution source. Totally three fecal samples were collected from Conservation and Research Center for Bear Species in Sichuan province (named S1 sample), Yunnan province (named Y1 sample) and Heilongjiang province (named H1 sample) in China respectively. All samples were collected from healthy animals (Table S1).

### DNA isolation, PCR amplification and MiSeq sequencing

Total genomic DNA was isolated from fecal samples using the Qiagen QIAamp Fast DNA Stool Mini Kit according to the manufacturer’s instructions. The concentration of isolated DNA was determined using a Nano-Drop 2000 spectrophotometer. PE amplicon libraries were constructed, and sequencing was performed using the Illumina MiSeq platform at Majorbio Bio-Pharm Technology Co., Ltd., Shanghai, China. Libraries with an insert size of 300 bp were constructed from our samples. Raw reads (101 bp in length for S1 sample, 151 bp in length for H1 and Y1 samples) were trimmed to remove low quality reads that contained ambiguous nucleotides or had a quality value lower than 20^22^. The raw metagenomic sequences are available at the NCBI Short Reads Archive under the accession number SRP079591.

### Identification of genes encoding HSDHs

Clean reads were assembled using the SOAPdenovo assembler at a range of Kmers (39–47). Default settings of SOAPdenovo were used for all the assemblies. The longest contig lengths, N50 and N90, were used to access the best assembly results. Then scaffold with a length over 500 bp were extracted and broken into contigs without gaps. Contigs were used for the prediction of open reading frames using the MetaGeneAnnotator. Predicted ORFs with length over 100 bp were used for the identification of target gene.

Local Blast programs analysis was performed to identify the 7α/7β-HSDHs encoding genes. The protein sequences of 7α-HSDH (EC 1.1.1.159) and 7β-HSDHs (EC 1.1.1.201) reported so far were used as models to anchor the homologous proteins. For each predicted gene, hits with E-value < 1e-5 were kept and sequences with more than 50% identity were chosen as candidate genes for further studies.

### Gene cloning, expression, and protein purification

Total genomic DNA was isolated from fecal samples using the Qiagen QIAamp Fast DNA Stool Mini Kit according to the manufacturer’s instructions. The genes encoding 7α- and 7β-HSDHs discovered by the process described above were amplified by polymerase chain reaction (PCR) using sequence specific primers (Table S2). The 7α- and 7β-HSDH encoding genes from *C. absonum* ATCC27555 were also cloned in order to compare their enzymatic characteristics in further steps of this study. The PCR products were cloned into the expression vector pGEX-6P-1 using *Bam*H I and *Xho* I. *E. coli* BL21 cells were transformed with the expression constructs respectively and grown in Luria Bertani medium containing 50 ug/mL ampicillin. Target gene expression was induced by adding isoproryl β-D-Thiogalactoside at a final concentration of 0.2 mM to exponentially growing cells at an OD_600_ of 0.8, at which time the culture temperature was shifted from 37 °C to 16 °C. After 12 h of culture at 16 °C, the cells were collected by centrifugation at 8,000 × *g* for 5 min at 4 °C. Recombination proteins were purified according to the manufacturer’s instructions, and PreScission protease was used to excise the GST tag from the recombinant proteins. The efficiency of PreScission protease were verified by SDS-PAGE.

### Measurement of enzyme activity and analysis of conversion products

The enzyme activity was determined spectrophotometrically at 340 nm and 25 °C by measuring oxidation of NADPH or reduction of NADP^+^. The standard assay mixture (2 mL) for the determination of 7α-HSDH activity assay: 0.5 mM TCDCA in 50 mM Tris-HCl buffer, pH 8.0, and 0.5 mM NADP^+^. For the determination of 7β-HSDH activity the mixture was: 0.5 mM TUDCA in 50 mM Tris-HCl, pH 8.0, and 0.5 mM NADP^+^. One unit of the activity is defined as the enzyme activity that reduces 1 μmol of NADP^+^ per minute under the assay conditions described above. To identify the bioconversion abilities of the recombination proteins, the reaction mixtures were monitored via high-performance liquid chromatography (HPLC). All reaction products were analyzed by UV detection at 205 nm using a mobile phase of acetonitrile-ammonium acetate (final ratio 42:58, pH 4.5) and a C18 column.

### Melting temperature characterization and essential gene prediction

Melting temperature was assessed using CD (222 nm, far UVCD) by following changes in the spectrum with increasing temperature from 20 °C to 95 °C. The software CDpal was used to analyze thermal denaturation data in order to obtain information on protein stability^23^. The two-state model and Autofit procedure were chosen to determine the melting temperature (Tm) of each protein samples. Each measurement was performed at least three times. The CEG (Cluster of Essential Genes) database was used to predict the essentiality of these genes^24^. The minimum value of matches in CEG database was selected as 6, and the match type was chosen as Gene_name.

### Kinetic analysis and enzyme thermal stability

The kinetic constants were determined by using varying concentrations of TCDCA or TUDCA for the enzyme assay reaction in 25 °C. Kinetic parameters (*K*_*m*_, *V*_*max*_ and *k*_*cat*_) were calculated with Microsoft Excel 2013 using the Michaelis–Menten equation. For the determination of the enzyme thermal stability of HSDH activities, the enzymes were incubated at 4, 25 and 37 °C for various time periods highest being 48 h (in 50 mM Tris-HCl buffer, pH 8.0). The remaining enzymatic activity was measured under standard conditions. All experiments were performed in at least triplicate using purified enzyme samples.

### Sequence alignment and phylogenetic analysis

Multiple amino acid sequence alignments of the newly discovered enzymes as well as known ones were carried out using the DNAMAN software. A phylogenetic tree of these enzymes was constructed using the MEGA5 software.

## Results

### Discovery, cloning and expression of HSDH genes

In total, 250,908,092 clean reads were generated with an average of 83,636,030 reads per sample. Statistical information of contigs is listed in Table S3. After searching the datasets consisting of the predicted ORFs, we successfully cloned and purified six full-length HSDH genes from the three fecal samples (Table 1): two 7α-HSDH encoding genes from S1, two 7α-HSDH encoding genes from H1, one 7α-HSDH and one 7β-HSDH encoding gene from sample Y1. The six HSDH genes discovered above and the 7α- and 7β-HSDH encoding genes from *C. absonum* ATCC27555 were overexpressed as GST-fused recombination proteins, and PreScission protease was used to excise the GST tag from the recombinant proteins. The quality of purified enzymes were verified by SDS-PAGE (Fig. S1).

### Verification of enzymatic function

**Figure 1:**
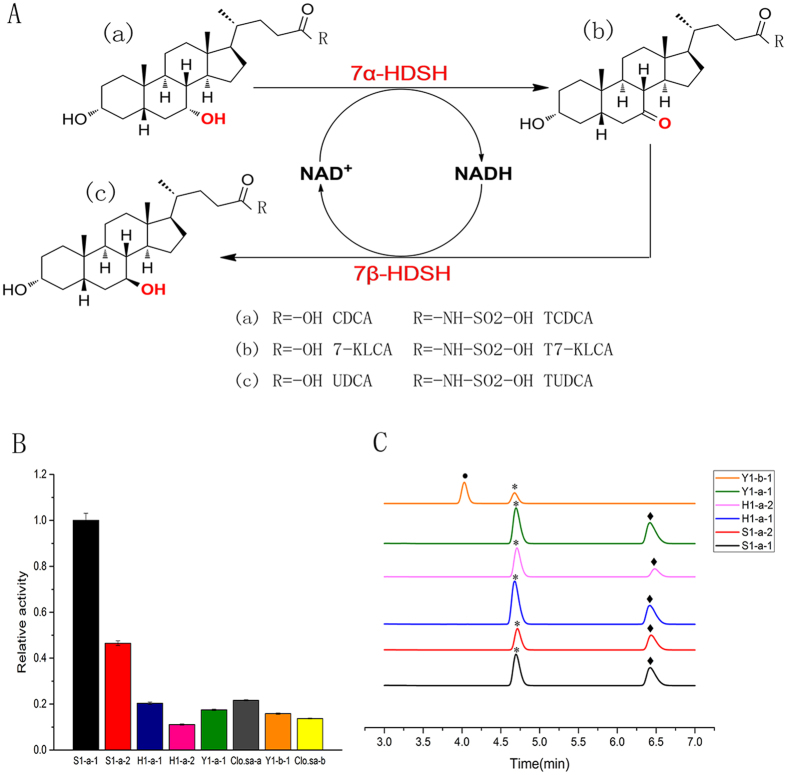
(**A**) Epimerization reaction from TCDCA to TUDCA, which is catalyzed by 7α-HSDH and 7β-HSDH enzymes. (**B**) Relative activities of 7α- and 7β-HSDH enzymes. Five 7α-HSDHs (S1-a-1, S1-a-2, H1-a-1, H1-a-2, and Y1-a-1) and one 7β-HSDH (Y1-b-1) discovered in the present study, together with one known 7α-HSDH (Clo.sa-a) and 7β-HSDH (Clo.sa-b) purified from *C. absonum* ATCC27555 were tested for enzymatic activity respectively. (**C**) HPLC analyze of bioconversion products of candidate 7α- and 7β-HSDH enzymes discovered in this study: •TUDCA, *T7-KLCA, ◆TCDCA.

We tested the eight proteins (five 7α-HSDHs and one 7β-HSDH discovered in the present study, and one known 7α-HSDH (name as Clo.sa-a) and 7β-HSDH (name as Clo.sa-b) purified from *C. absonum* ATCC27555) for enzymatic activity (Fig. 1B). All the proteins discovered in the present study showed enzymatic activity towards their corresponding substrate, suggesting that the candidate genes predicted by our metagenomic strategy are enriched in enzymes with relevant activities. Comparing the activity of the discovered five 7α-HSDHs to the known one purified from *C. absonum* ATCC27555, protein S1-a-1 and S1-a-2 showed five-fold and two-fold enzymatic activity of the known one. Protein H1-a-1 and Y1-a-1 displayed an enzymatic activity generally comparable to the known one. The newly discovered 7β-HSDH presented an enzymatic activity equal to the one purified from *C. absonum* ATCC27555.

To identify the conversion ability of the five 7α-HSDHs and one 7β-HSDH, the conversion products of the purified enzymes were subjected to HPLC (Fig. 1C). Both the oxidation and reduction reaction products, corresponding to the substrate TCDCA (catalyzed by 7α-HSDH) and TUDCA (catalyzed by 7β-HSDH), were detected by HPLC. HPLC results indicated the interconversion between TCDCA (or TUDCA) and T7-KLCA, which clearly demonstrated the conversion ability of the five 7α-HSDHs and one 7β-HSDH.

### Homology analysis of the enzymes

The amino acid sequences of 7α-HSDHs from *C. sardiniense* (Clo.sa-a, Genebank No. AET80685), *C. sordellii* (Clo.so-a, Genebank No. AAA53556), *C. scindens* (Clo.sc-a, Genebank No. AAB61151), *B. fragilis* (Bac-fr-a, Genebank No. AAD49430) and *E.coli* (Esc.co-a, Genebank No. P0AET8) were used for alignment with the discovered ones, and there was on average a 62.48% identy at the deduced amino acid level. In addition, the amino acid sequence of Y1-b-1 was compared to known 7β-HSDH sequences from *C. sardiniense* (Clo.sa-a, Genebank No. AET80684), *C. aerofaciens* (Col.ae-b Genebank No. ZP0177306), *R.torques* (Rum.to-b Genebank No. CBL26204.) and *R.gnavus* (Rum.gn-b, Genebank No. ZP_02041813). Alignment of the amino acid sequences indicated high sequence identity of Y1-b-1 to Col.ae-b (75.28% homology), Rum.to-b (81.65% homology) and Rum.gn-b (74.53% homology), but relatively low homology to Col.ae-b (42.32% homology). The amino acid sequence similarities of the 7α-and 7β-HSDHs also suggested that they all belongs to conserved domains of the short-chain dehydrogenase (SDR) family. It is known that the SDRs share very low homology and sequence identity. However, the sequence alignment clearly showed conserved domains in the SDR primary structure, including the N-terminal G-X-G cofactor-binding motif (around position 18 in 7α-HSDHs, and position 22 in 7β-HSDHs)^25^. Further, the N-N-X-G motif (around position 90), which is important for coenzyme binding^26^, is found in 7α-HSDHs, and singular Ser-143, Tyr-158 and Lys-162 (the numbers refers to Y1-b-1), putative active site residues that may comprise the catalytic triad of SDRs^26^, are found in 7β-HSDHs.

**Figure 2:**
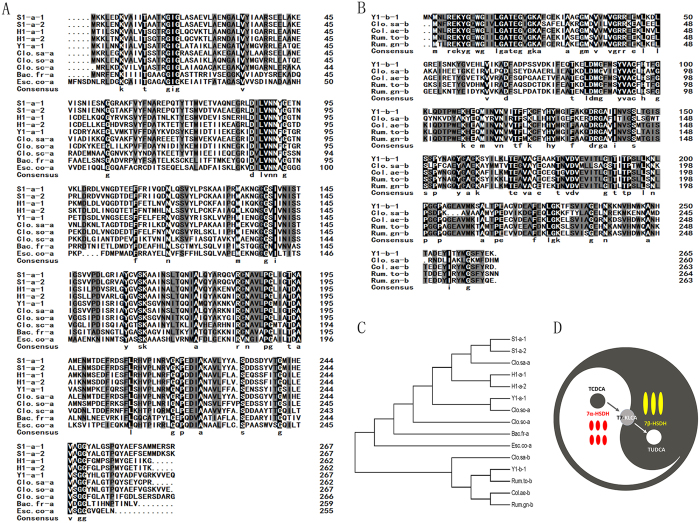
(**A**) Amino acid sequence alignment of 7α-HSDHs from this study and the other selected 7α-HSDHs. The amino acid sequences of 7α-HSDHs from *C. sardiniense* (Clo.sa-a, Genebank No. AET80685), *C. sordellii* (Clo.so-a, Genebank No. AAA53556), *C. scindens* (Clo.sc-a, Genebank No. AAB61151), *B. fragilis* (Bac-fr-a, Genebank No. AAD49430) and *E.coli* (Esc.co-a, Genebank No. P0AET8) were used for alignment with the discovered 7α-HSDHs (S1-a-1, S1-a-2, H1-a-1, H1-a-2, and Y1-a-1) in this study. (**B**) Amino acid sequence alignment of 7β-HSDHs from this study and the other selected 7β-HSDHs. The amino acid sequence of Y1-b-1 was compared to known 7β-HSDH sequences from *C. sardiniense* (Clo.sa-a, Genebank No. AET80684), *C. aerofaciens* (Col.ae-b Genebank No. ZP0177306), *R.torques* (Rum.to-b Genebank No. CBL26204.) and *R.gnavus* (Rum.gn-b, Genebank No. ZP_02041813). (**C**) Phylogenetic tree based on alignment of 7α-HSDH and 7β-HSDH protein sequences. (**D**) The cooperation biotransformation of TCDCA to TUDCA by 7α-HSDH and 7β-HSDH enzymes in gut microbiota of black bears.

The evolutionary tree based on the alignment represented in Fig. 2A,B is shown in Fig. 2C. The HSDHs are classified by function rather than species. The 7α-HSDHs from the present study and *C. sardiniense, C. sordellii, C. scindens, B. fragilis* and *E.coli* belong to the same subgroup. Y1-b-1 show a much closer relationship to 7β-HSDH from *R.torques*, and together with other 7β-HSDHs from *C. sardiniense, C. aerofaciens* and *R.gnavus* clustered into another subgroup.

### Characterization of enzymatic properties

The melting temperatures (Tm) and essentialities of the five 7α-HSDHs and one 7β-HSDH are shown in Table 1. CD data were analyzed by CDpal and the melting temperature curves of each protein sample are presented in Figs S2 to S7. Kinetic parameters of each enzyme, including *K*_*m*_, *V*_*max*_ and *k*_*cat*_ values for their corresponding substrates, were determined by using the Michaelis–Menten equation (Table 2). In general, 7β-HSDH Y1-b-1 had the lowest *T*_*m*_ compared the other five 7α-HSDHs, and H1-a-1 had the highest *T*_*m*_ among the six enzymes discovered in this study. As for *V*_*max*_ values and catalytic efficiencies (*k*_*cat*_/*K*_*m*_), S1-a-1 and S1-a-2 present a significantly higher *V*_*max*_ and better catalytic efficiencies compared to Clo.sa-a, the 7α-HSDH from *C. sardiniense*, and Y1-b-1 presents an equivalent *V*_*max*_ value and catalytic efficiency compared to Clo.sa-b, the 7β-HSDH from *C. sardiniense*. All the enzymes discovered in the present study were shown to be essential genes and are conserved in multiple bacterial species.

**Figure 3:**
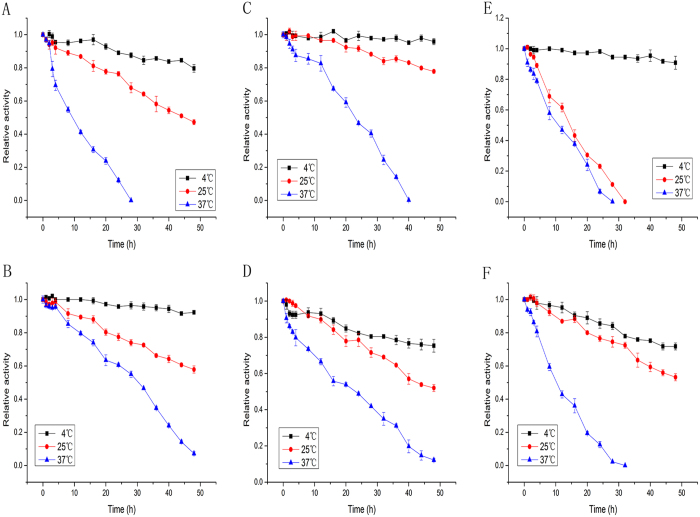
(**A**,**B**) Thermostability of the purified 7α-HSDHs S1-a-1 and S1-a-2 from S1 sample. (**C**,**D**) Thermostability of the purified 7α-HSDHs H1-a-1 and H1-a-2 from H1 sample. (**E**,**F**) Thermostability of the purified 7α-HSDH Y1-a-1 and 7β-HSDH Y1-b-1 from Y1 sample.

The thermostability of the purified five 7α-HSDHs and one 7β-HSDH was examined at temperatures of 4, 25 and 37 °C (Fig. 3A–F). For S1-a-2, H1-a-1, and Y1-a-1, no significant loss of activity was observed during incubation at 4 °C for 48 h. However after 48 h, the residual activity of S1-a-1, H1-a-2, and Y1-b-1 were about 80%, 75%, and 71%, respectively. All the enzymes presented at least half of the residual activity after 48 h at 25 °C except Y1-a-1, which showed no detectable activity after 32 h at 25 °C. The residual activities of S1-a-2, H1-a-1, and H1-a-2 were still about 62%, 60%, and 52%, respectively, after heat treatment at 37 °C for 24 h, while after 48 h, only S1-a-2 and H1-a-2 showed remaining activities of about 7% and 12%, respectively.

## Discussion

7α-HSDH and 7β-HSDH catalyze the reversible, stereospecific oxidation/reduction of the 7α/7β-hydroxy group of bile acids and play a key role in 7-epimerization. Molecular cloning of genes encoding 7α-HSDH and 7β-HSDH enzymes will help to understand the genetic organization and regulation of metabolic pathways of bile salt-modifying bacteria. From a practical point of view, they may become useful enzymes for the bio-transformation of TCDCA to TUDCA, an important pharmaceutical agent. To the best of our knowledge, five genes encoding 7α-HSDHs^14^,^15^,^16^,^17^,^18^ and four genes encoding 7β-HSDHs^18^,^19^,^20^,^21^ have been cloned so far, but only few of them were discovered from fecal samples. In this work, high-throughput metagenomic sequencing has provided a powerful alternative to discovering HSDHs from the gut microbiota, and we successfully identified and cloned five full-length 7α-HSDHs and one 7β-HSDH encoding gene, and their enzymatic properties were characterized.

Four enzymes discovered in this study present good enzymatic properties. The 7α-HSDHs, S1-a-1 and S1-a-2, showed five-fold and two-fold higher enzymatic activities, respectively, compared to that of Clo.sa-a, and H1-a-1 showed an enzymatic activity equivalent to that of Clo.sa-a. S1-a-1 and S1-a-2 also presented much better catalytic efficiencies, which were ten-fold and two-fold higher than that of Clo.sa-a, respectively. The thermostability of these three enzymes is roughly the same as that of Clo.sa-a (Fig. S8). The 7β-HSDH, Y1-b-1, had an enzymatic activity and catalytic efficiency equivalent to that of Clo.sa-b. Thermostability of Y1-b-1, however was much higher compared to that of Clo.sa-b (Fig. S8) and Rum.to-b^21^, the latest 7β-HSDH reported so far. For example, the residual activity of Clo.sa-b was completely inactivated at 37 °C after 12 h, Rum.to-b present a 20% residual activity after the same treatment, while Y1-b-1 was still about 90% of the activity remained after 12 h at 37 °C. Since those enzymes were from gut microbiota, intestinal microenvironment may be one of factors for the evolutionary for their good thermal stabilities.

Furthermore, many putative partial 7α-HSDHs and 7β-HSDHs encoding gene fragments were also discovered from the three fecal samples (Table S4). These data suggest a cooperation of the gut microbiota in black bears for biotransformation of TUDCA (Fig. 2D). According to our gene identification settings, there were no conditional putative 7β-HSDH encoding genes found in sample H1. Since the sequence identity in pair-wise comparisons between diff erent SDR enzymes is typically 15–30%, and compared to 7β-HSDHs from different species reported so far, a low residue identity of 40% was shown. There may be more 7β-HSDH candidates hidden in the metagenomic sequences, which share a lower residue identity with the known enzymes, and in future, deeper data mining and enzyme function determination assays are necessary to verify this corollary.

In summary, we have successfully discovered five new 7α-HSDHs and one new 7β-HSDH from the gut microbiota of black bears by a metagenomic approach, thereby expanding the natural enzyme bank of HSDHs. Moreover, detailed functional characterization indicated that four enzymes could be promising candidates for further applications in the bioconversion of TCDCA to TUDCA and the epimerization reaction of bile acids at C-7 position. Although this work focused on the identification and validation of 7α-HSDH and 7β-HSDH, these data sets provide an extensive resource for the discovery of other novel enzymes in the gut microbiome of black bears, and the general approach presented here will be applicable to another environmental microbiota.

## Additional Information

**How to cite this article**: Song, C. *et al*. Discovery of tauroursodeoxycholic acid biotransformation enzymes from the gut microbiome of black bears using metagenomics. *Sci. Rep.* **7**, 45495; doi: 10.1038/srep45495 (2017).

**Publisher's note:** Springer Nature remains neutral with regard to jurisdictional claims in published maps and institutional affiliations.

## Supplementary Material

Supplementary Information

## Figures and Tables

**Table 1:** Information of melting temperatures and essentialities of the 7α-HSDHs and 7β-HSDH discovered from fecal samples.

Enzyme Name	Sample ID	Putative Function	Tm (°C)	Essentiality
S1-a-1	S1 Sample	7α-HSDH	64.42644	essential
S1-a-2	S1 Sample	7α-HSDH	66.05438	essential
H1-a-1	H1 Sample	7α-HSDH	68.72841	essential
H1-a-2	H1 Sample	7α-HSDH	51.24016	essential
Y1-a-1	Y1 Sample	7α-HSDH	61.36976	essential
Y1-b-1	Y1 Sample	7β-HSDH	48.42976	essential

**Table 2:** Summary of kinetic parameters of the purified enzymes.

Enzyme	Substrate	*K*_*m*_ (mM)	*V*_*max*_ (U/mg)	*k*_*cat*_ (S^−1^)	*k*_*cat*_/*K*_*m*_
S1-a-1	TCDCA	0.132	369.230	236.538	1791.954
S1-a-2	TCDCA	0.263	177.966	56.285	214.011
H1-a-1	TCDCA	0.390	70.222	10.400	26.666
H1-a-2	TCDCA	0.695	32.627	2.280	3.280
Y1-a-1	TCDCA	0.556	31.250	18.571	33.401
Clo.sa-a	TCDCA	0.241	22.470	40.103	166.402
Y1-b-1	TUDCA	6.153	65.116	12.558	2.040
Clo.sa-b	TUDCA	3.062	52.801	7.767	2.536
