# Supplementary material for: Discovery of tauroursodeoxycholic acid biotransformation enzymes from the gut microbiome of black bears using metagenomics
Source: Sci Rep. 2017 Apr 24;7:45495. doi: 10.1038/srep45495 (PMC5402301; doi:10.1038/srep45495)
Supplement: Supplementary Information [file srep45495-s1.doc]

**Supplementary materials:**

Discovery of tauroursodeoxycholic acid biotransformation enzymes from the gut microbiome of black bears using metagenomics

Can Song^1^, Bochu Wang^1^,*, Jun Tan^2^,*, Liancai Zhu^1^, Deshuai Lou^1^

^1^ Key Laboratory of Biorheological Science and Technology (Chongqing University), Ministry of Education, College of Bioengineering, Chongqing University, Chongqing 400030, China

^2^ Chongqing Key Laboratory of Medicinal Resources in the Three Gorges Reservoir Region, School of Biological & Chemical engineering, Chongqing University of Education, Chongqing 400067, China

*Corresponding authors: [wangbc2000@126.com](mailto:wangbc2000@126.com) (Bochu Wang) and [tanjunmail@126.com](mailto:tanjunmail@126.com) (Jun Tan); Telephone: +86-023-65112840; Fax: +86-023-65112877.

Table S1. Information on the black bears used in this study.

| **Sample ID** | **Sex** | **Age** | **Health condition** | **Collection**  **time** |
| --- | --- | --- | --- | --- |
| S1 | male | adult | healthy | Jul. 2014 |
| Y1 | male | adult | healthy | Dec. 2014 |
| H1 | male | adult | healthy | Jan. 2015 |

※ Adult refers to an individual more than 8 years of age.

Table S2. Primers to clone the 7α/7β-HSDH encoding genes discovered in this study.

| **Primers** | **Sequences (5’→3’)** |
| --- | --- |
| S1-a-1-f | ATGAAAAAGTTAGAAGATAAAGTAG |
| S1-a-1-r | CTATCTACTTCTCTCCATCATTG |
| S1-a-2-f | ATGAGAAAACTTGAAAATAAGGTAG |
| S1-a-2-r | TTACTTACTTTTATCCATCATCTCTG |
| H1-a-1-f | ATGACCATTCTTTCTAATAAAGTAGCACTT |
| H1-a-1-r | TTAACCCTTGATAATCTCACCATACATG |
| H1-a-2-f | ATGAAAAAATTAACAAATAAAGTTGCCCT |
| H1-a-2-r | TTATTTTGTAATTGTTTCACCGTACATT |
| Y1-a-1-f | ATGAAAATTTTAAATAACAAAATAGCTTTAGT |
| Y1-a-1-r | TTAATCTTGCTCAACAACTTTACGT |
| Y1-b-1-f | ATGAATATGAATTTAAGAGAAAAATATGGAG |
| Y1-b-1-r | TTATTTCTCATAGAAAGACCCCATATAT |

Table S3. Summary for the metagenomic sequencing, assembly and annotation.

| **Category** | **S1** | **H1** | **Y1** |
| --- | --- | --- | --- |
| Raw reads | 93964956 | 61697850 | 103074898 |
| Clean reads | 90735525 | 59632218 | 100540349 |
| Assemble contigs | 131415 | 39845 | 54252 |
| Contig max (bp) | 202749 | 192228 | 118410 |
| Contig min (bp) | 300 | 500 | 500 |
| N50 (bp) | 1476 | 3392 | 1903 |
| N90 (bp) | 426 | 711 | 569 |
| Predicted ORFs | 210990 | 96928 | 92200 |

Table S4. Abundances of putative 7α- and 7β-HSDHs (including partial and full segments) discovered in each sample (E-value < 1e-5, identity > 50%).

| Enzyme | S1 | H1 | Y1 |
| --- | --- | --- | --- |
| 7α-HSDH | 18 | 8 | 12 |
| 7β-HSDH | 6 | ND | 4 |


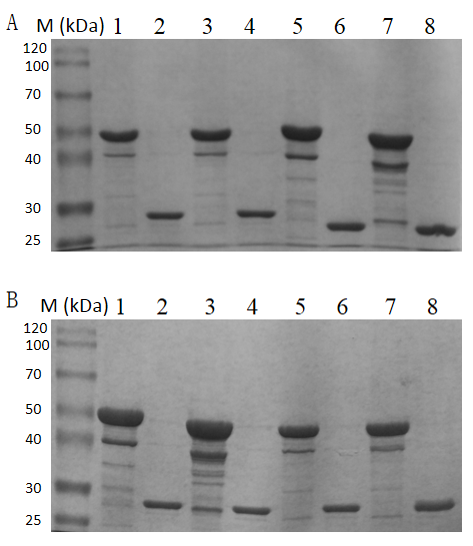


Figure S1. (A) and (B) SDS-PAGE analysis of the purified enzymes. (A) Lane M, Blue plus II protein marker (Transgen, China); Lane 1, Glutathione-sepharose beads bound to GST-tagged protein S1-a-1; Lane 2, the purified protein S1-a-1 without GST tag; Lane 3, Glutathione-sepharose beads bound to GST-tagged protein S1-a-2; Lane 4, the purified protein S1-a-2 without GST tag; Lane 5, Glutathione-sepharose beads bound to GST-tagged protein H1-a-1; Lane 6, the purified protein H1-a-1 without GST tag; Lane 7, Glutathione-sepharose beads bound to GST-tagged protein H1-a-2; Lane 8, the purified protein H1-a-2 without GST tag. (B) Lane M, Blue plus II protein marker (Transgen, China); Lane 1, Glutathione-sepharose beads bound to GST-tagged protein Y1-a-1; Lane 2, the purified protein Y1-a-1 without GST tag; Lane 3, Glutathione-sepharose beads bound to GST-tagged protein Y1-b-1; Lane 4, the purified protein Y1-b-1 without GST tag; Lane 5, Glutathione-sepharose beads bound to GST-tagged protein Clo.sa-a; Lane 6, the purified protein Clo.sa-a without GST tag; Lane 7, Glutathione-sepharose beads bound to GST-tagged protein Clo.sa-b; Lane 8, the purified protein Clo.sa-b without GST tag.


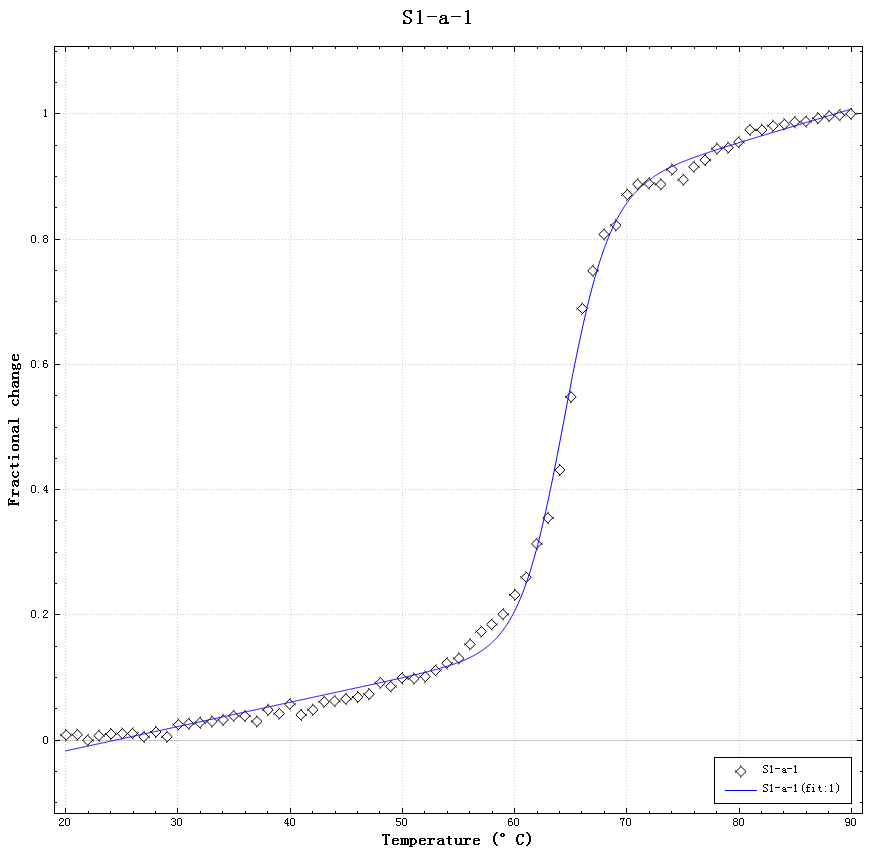


Figure S2. Thermal denaturation curves of S1-a-1.


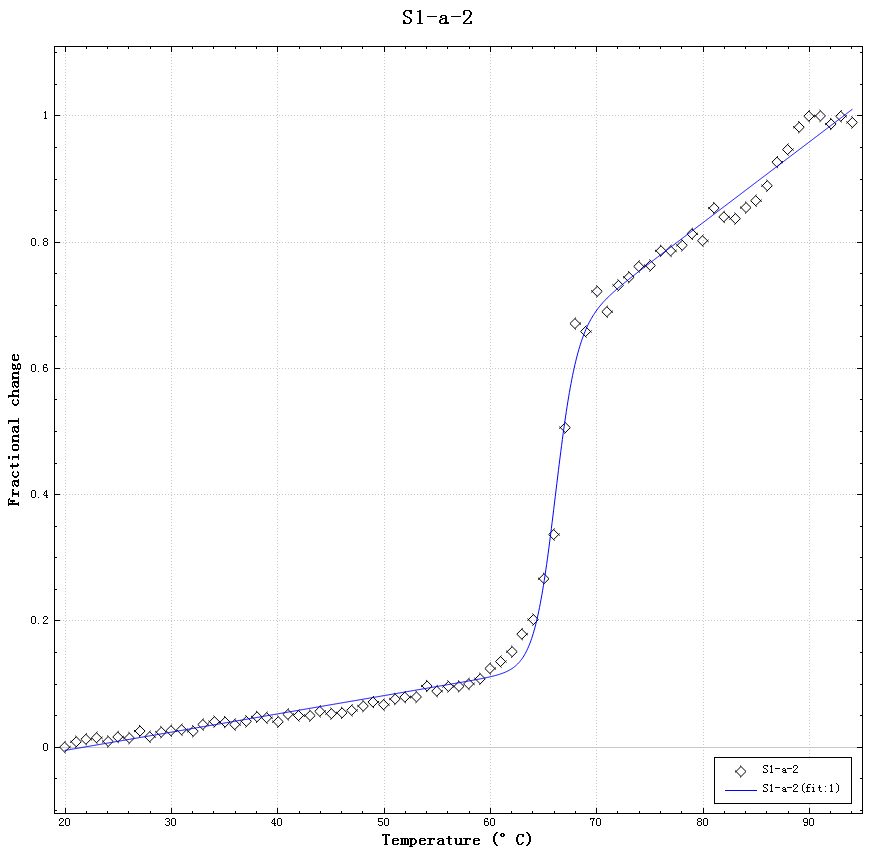


Figure S3. Thermal denaturation curves of S1-a-2.


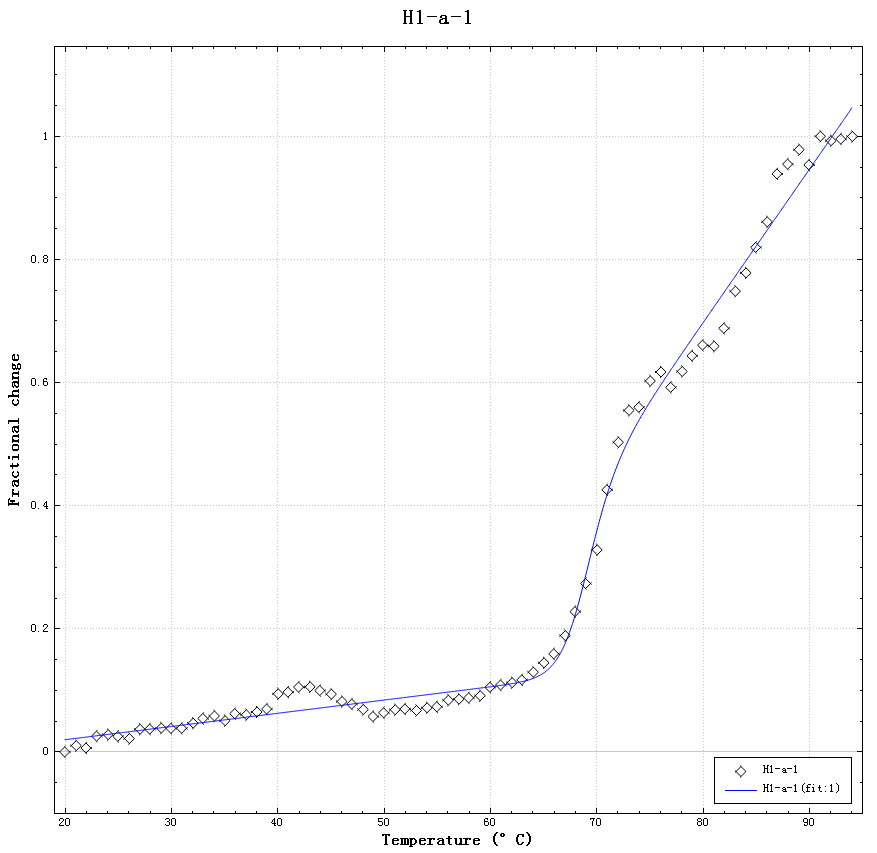


Figure S4. Thermal denaturation curves of H1-a-1.


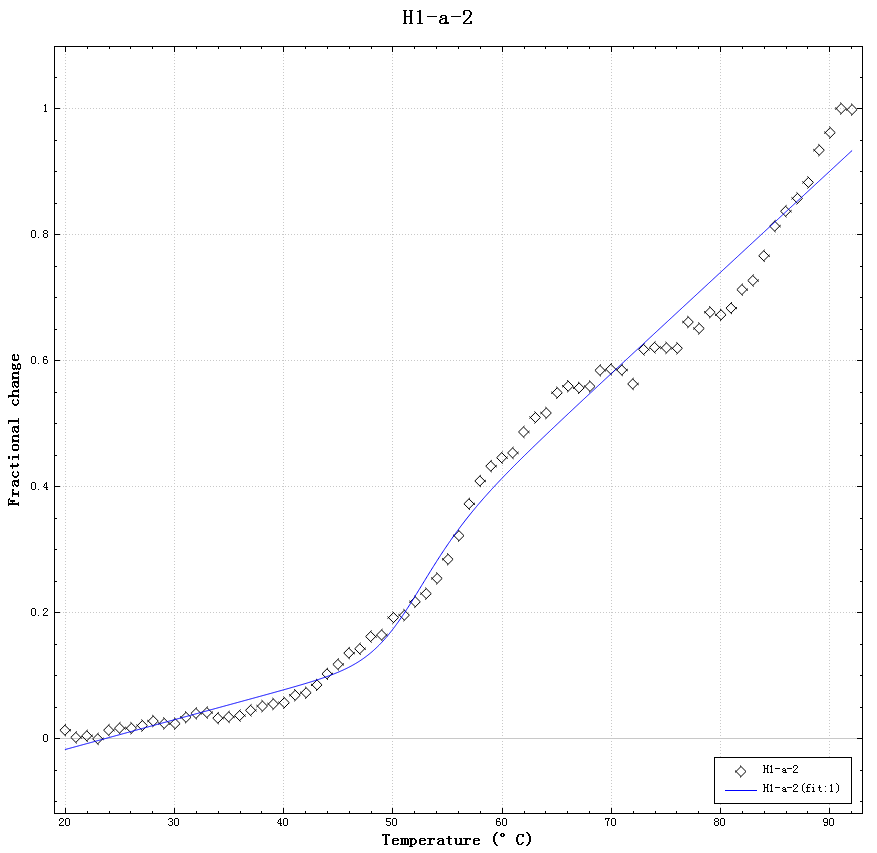


Figure S5. Thermal denaturation curves of H1-a-1.


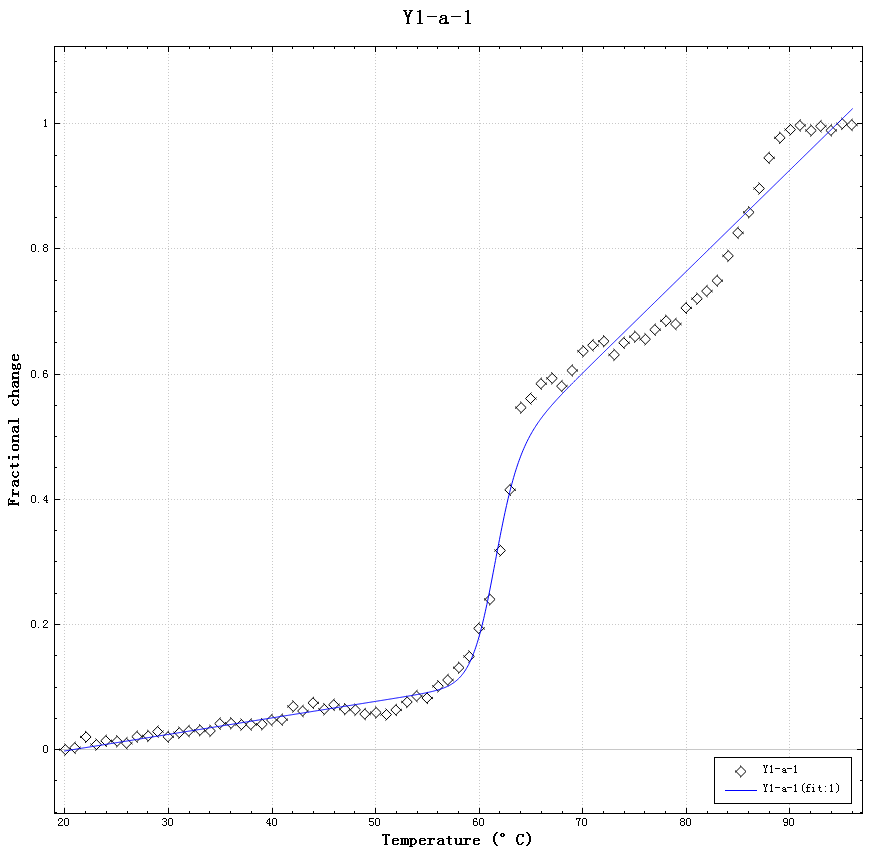


Figure S6. Thermal denaturation curves of Y1-a-1.


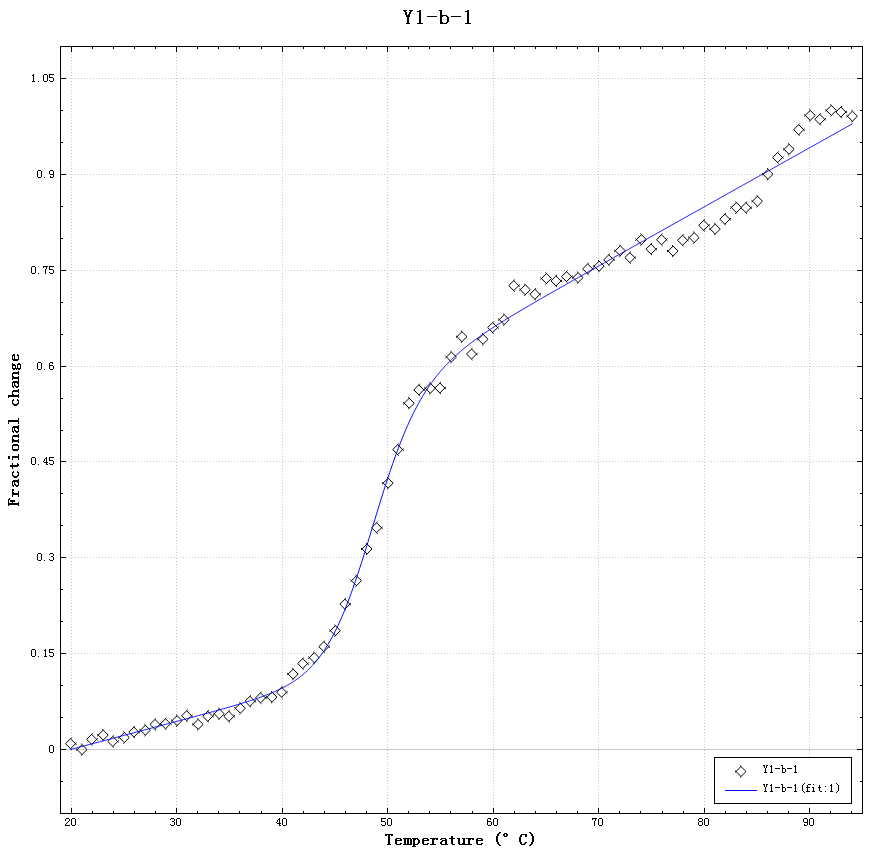


Figure S7. Thermal denaturation curves of Y1-b-1.


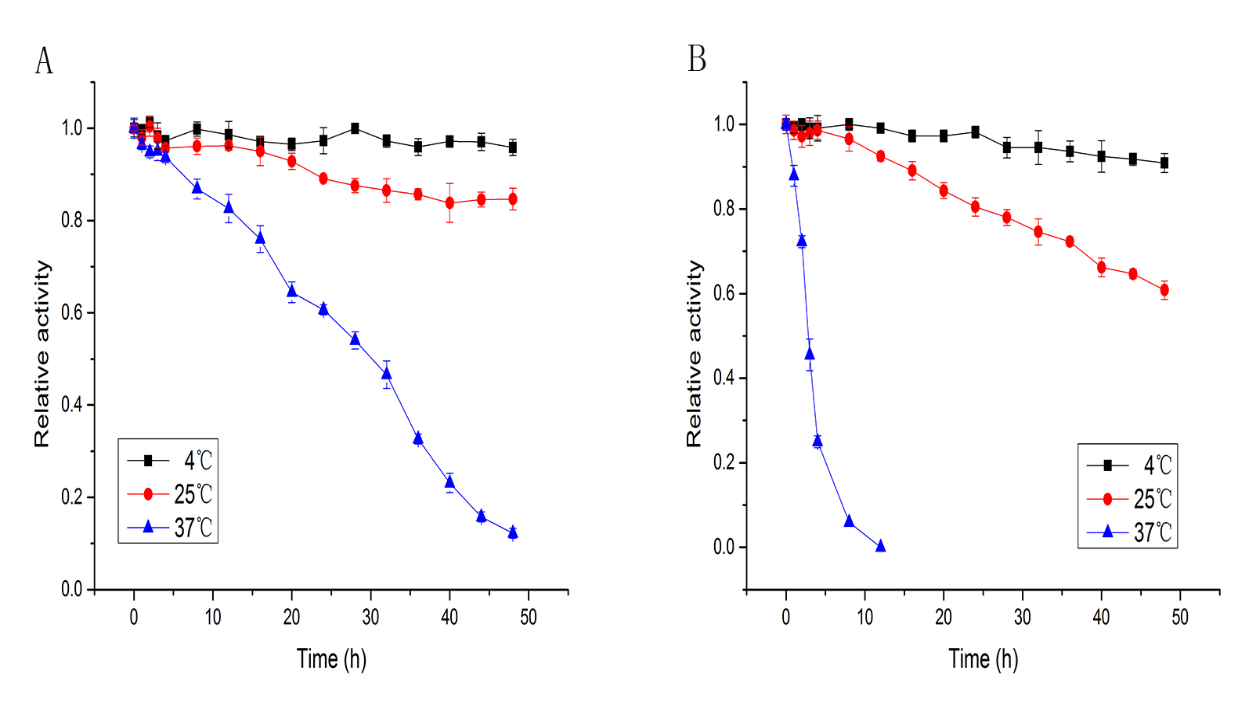


Figure S8. Thermostability of the purified 7α-HSDH Clo.sa-a (A) and 7β-HSDH Clo.sa-b (B) from *C. absonum* ATCC27555.
